# Supplementary material for: Increased aperiodic gamma power in young boys with Fragile X Syndrome is associated with better language ability
Source: Mol Autism. 2021 Feb 25;12:17. doi: 10.1186/s13229-021-00425-x (PMC7908768; doi:10.1186/s13229-021-00425-x)
Supplement: Supplementary file 1 — Additional file 1. Supplemental Figures and Tables. [file 13229_2021_425_MOESM1_ESM.pdf]

Supplemental Figure 1. FOOOF parameterization of power spectra. FOOOF estimated aperiodic (blue) and periodic (red) components are shown for each group. Actual averaged power spectra shown in green.

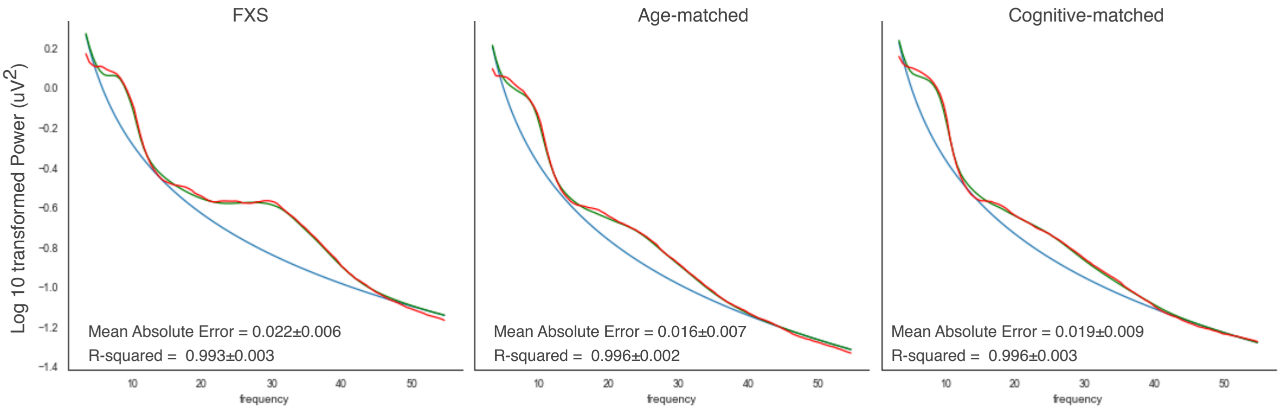

Supplemental Figure 2. Individual Plots of (A) Aperiodic and (B) Aperiodic-Adjusted power spectra.

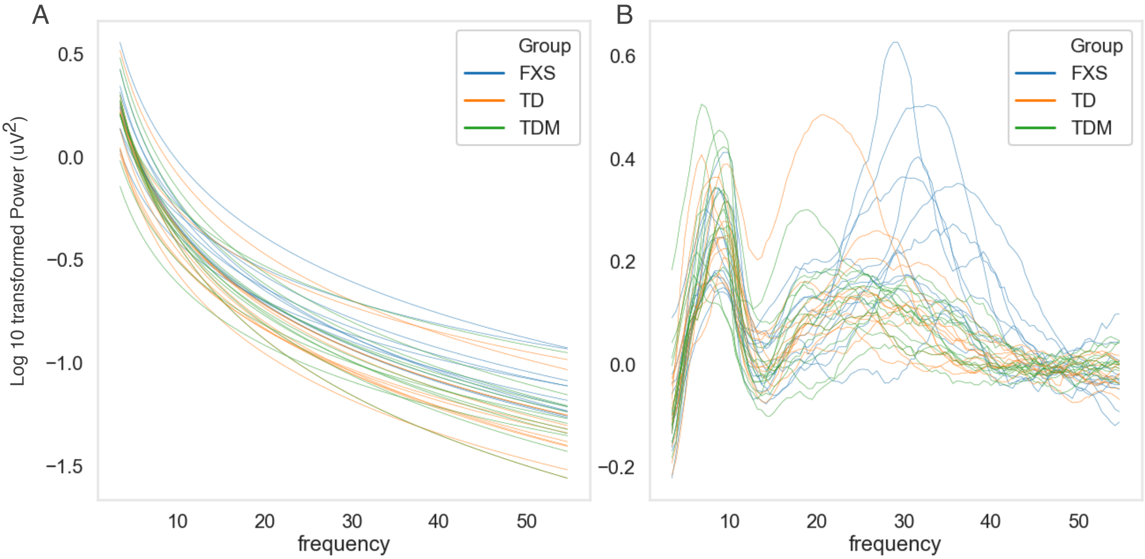

Supplemental Figure 3. Plots of Aperiodic and Aperiodic-Adjusted power spectra for all regions of interest

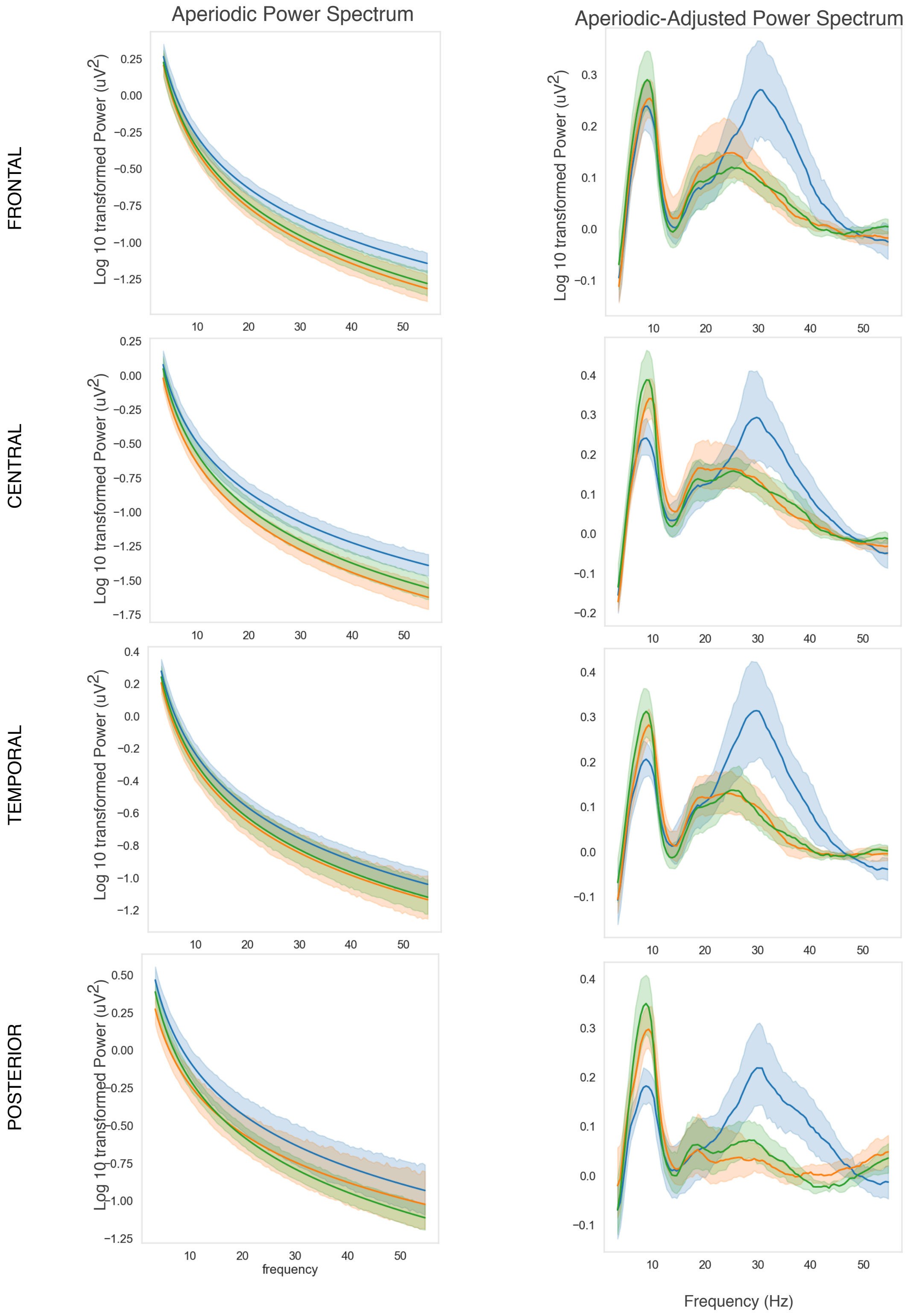

Supplemental Figure 4. Association of Age and power spectra measures

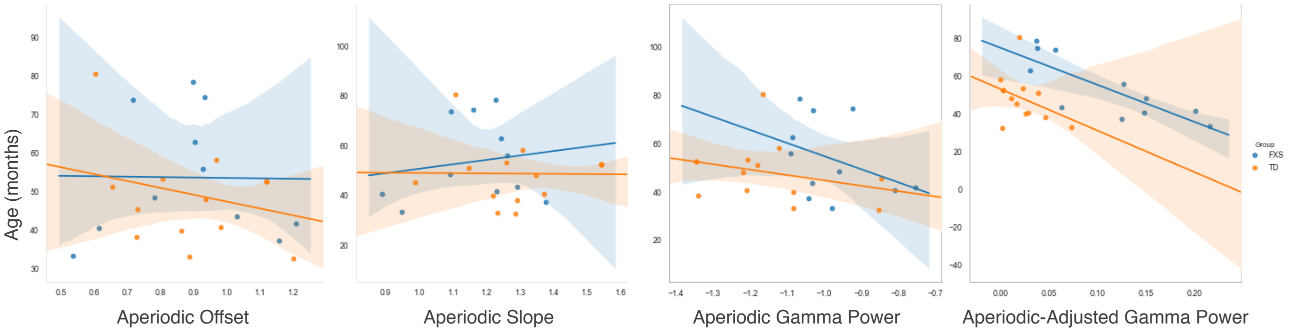

Supplemental Figure 5. Correlation graphs of additional clinical measures

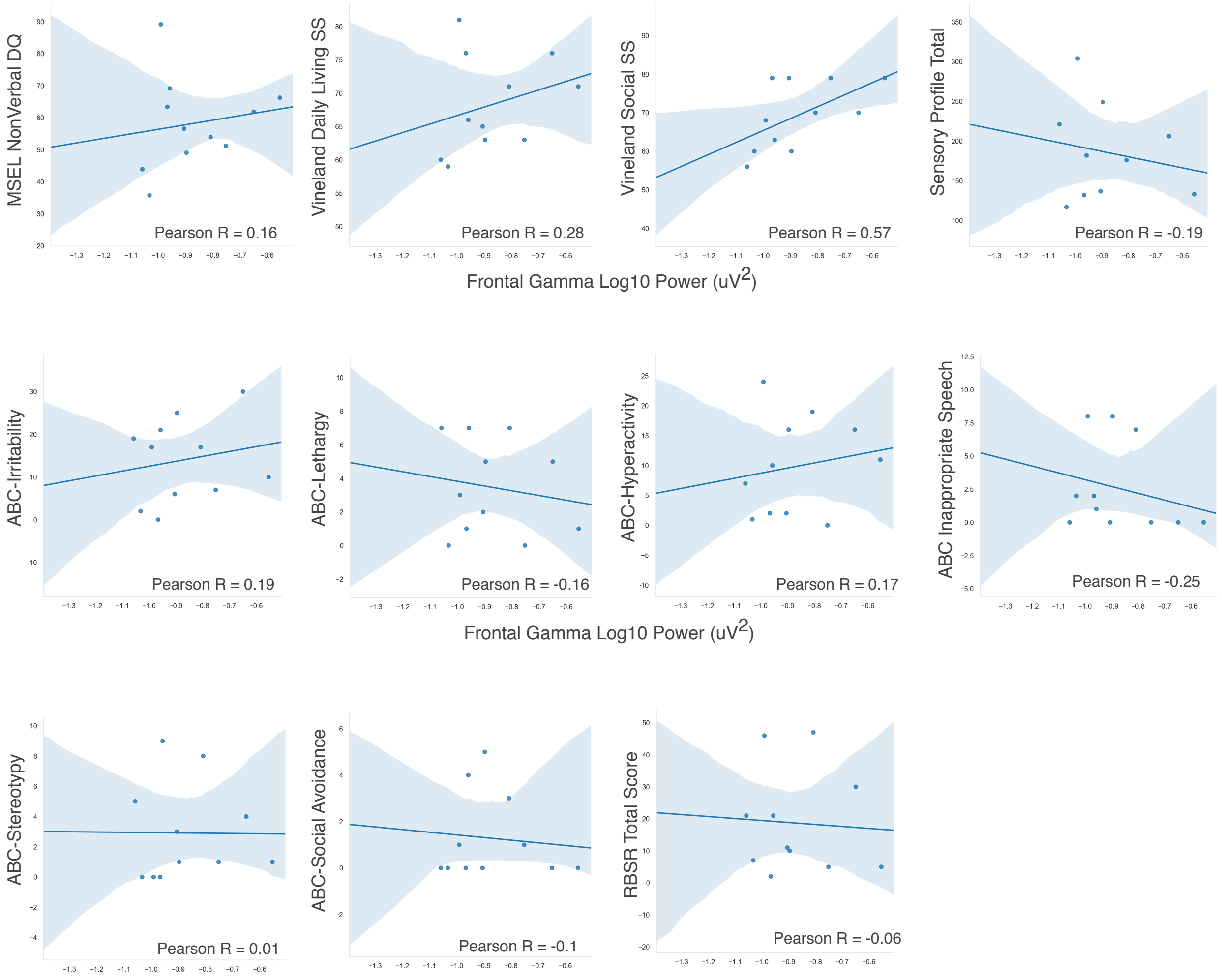

Supplemental Table 1. Aperiodic-adjusted power spectra clusters

	FXS vs Age-Matched		FXS vs Cognitive-Matched	
Region of Interest	Frequency Range (Hz)	Cluster p-value	Frequency Range (Hz)	Cluster p-value
Frontal	**28.8-41.0**	**0.003**	**25.9-43.0**	**0.005**
Central	6.8-10.3	0.11	5.4-8.8	0.137
Central	**26.4-38.1**	**0.016**	**25.9-36.1**	**0.04**
Central	-	-	50.8-52.7	0.36
Temporal	6.8-9.8	0.13	5.4-8.3	0.18
Temporal	**23.4-43.0**	**0.002**	**24.4-43.0**	**0.001**
Temporal	48.3-53.2	0.14	**47.4-53.2**	**0.04**
Posterior	4.9-9.3	0.08	4.4-8.8	0.07
Posterior	**22.9-43.0**	**0.002**	**24.4-45.4**	**0.02**
Posterior	49.3-53.2	0.16	50.8-52.2	0.32
